# Exploring the effects of missense mutations on protein thermodynamics through structure-based approaches: findings from the CAGI6 challenges

**DOI:** 10.1007/s00439-023-02623-4

**Published:** 2024-01-16

**Authors:** Carlos H. M. Rodrigues, Stephanie Portelli, David B. Ascher

**Affiliations:** 1 Computational Biology and Clinical Informatics, Baker Heart and Diabetes Institute, Melbourne, VIC 3004 Australia; 2 School of Chemistry and Molecular Biosciences, University of Queensland, St Lucia, QLD 4072 Australia

## Abstract

Missense mutations are known contributors to diverse genetic disorders, due to their subtle, single amino acid changes imparted on the resultant protein. Because of this, understanding the impact of these mutations on protein stability and function is crucial for unravelling disease mechanisms and developing targeted therapies. The Critical Assessment of Genome Interpretation (CAGI) provides a valuable platform for benchmarking state-of-the-art computational methods in predicting the impact of disease-related mutations on protein thermodynamics. Here we report the performance of our comprehensive platform of structure-based computational approaches to evaluate mutations impacting protein structure and function on 3 challenges from CAGI6: Calmodulin, MAPK1 and MAPK3. Our stability predictors have achieved correlations of up to 0.74 and AUCs of 1 when predicting changes in ΔΔ*G* for MAPK1 and MAPK3, respectively, and AUC of up to 0.75 in the Calmodulin challenge. Overall, our study highlights the importance of structure-based approaches in understanding the effects of missense mutations on protein thermodynamics. The results obtained from the CAGI6 challenges contribute to the ongoing efforts to enhance our understanding of disease mechanisms and facilitate the development of personalised medicine approaches.

**Supplementary Information:**

The online version contains supplementary material available at 10.1007/s00439-023-02623-4.

## Introduction

The sequencing of the first human genome over two decades ago has resulted in the efficient identification of genetic variation, however, characterising this into tangible, clinically-applicable information still remains a challenge. Different sequence-based tools, like SIFT (Sorting Intolerant From Tolerant) (Ng and Henikoff 2003) and Polyphen-2 (Adzhubei et al. 2013) have been widely used to assess the deleteriousness of a clinically observed mutation. These, similar to other sequence-based predictors CADD (Rentzsch et al. 2019), PROVEAN (Choi and Chan 2015) and SNAP2 (Hecht et al. 2015), are based on conservation trends acquired across multiple sequence alignments, which, despite highlighting potential functional effects, cannot predict changes on protein thermodynamic stability, ΔΔ*G*. Stability change predictions are especially important because they directly quantify the degree of effect on the protein fold, which had been evolutionarily optimised to serve a specific function (Stefl et al. 2013). Due to this, predictions of protein stability change tend to serve as a proxy for overall effects on protein fitness (Boucher et al. 2016), and hence offer insights into predispositions to disease.

Numerous predictors of protein stability change upon mutation have been developed, based on either statistical, empirical or machine-learning methods. Statistical methods include SDM (Site-Directed Mutator) (Worth et al. 2011) and SDM2 (Pandurangan et al. 2017) which use environment-specific amino-acid substitution matrices that quantify the probability of specific mutations to occur. Other statistical-based methods include DDGun (Montanucci et al. 2022), which linearly accounts for both sequence-based and structure-based features, and PoPMuSiC-2.1 (Dehouck et al. 2011), which is based on solvent accessibility parameters. Empirical approaches include Rosetta (Kellogg et al. 2011) and FoldX (Guerois et al. 2002), both of which account for intramolecular effects imparted by mutations, while CUPSAT (Parthiban et al. 2006) uses atom potentials and torsion angles representing the residue environment. Machine learning-based predictors, on the other hand, have been trained and validated on experimental ΔΔ*G* values and structural data, and include our tools: mCSM (Pires et al. 2014a), DynaMut (Rodrigues et al. 2018), DynaMut2 (Rodrigues et al. 2021) and DDMut (Zhou et al. 2023). Other machine learning-based tools encompassing include I-Mutant 3.0 (Capriotti et al. 2008), which relies on either structure or sequence, PROST (Iqbal et al. 2022) and SAAFEC-SEQ (Li et al. 2021), which are based on protein sequence, and MAESTRO (Laimer et al. 2015), which is based on multi-agent techniques. Some methods like DUET (Pires et al. 2014b) combine predictors offering different predictive methods, in this case statistical (SDM) and machine learning (mCSM). These tools have proven useful for the identification of disease associated (Serghini et al. 2023; Jessen-Howard et al. 2023; Al-Jarf et al. 2023; Stephenson et al. 2022; Karmakar et al. 2022; Hildebrand et al. 2020; Parthasarathy et al. 2022; Soardi et al. 2017; Andrews et al. 2018; Casey et al. 2017; Jafri et al. 2015) and drug resistance (Portelli et al. 2023a; Zhou et al. 2021; Karmakar et al. 2020, 2019, 2018; Vedithi et al. 2018, 2020) variants. Interestingly, they have been shown to perform as accurately using 3D models as with experimental structures (Iqbal et al. 2021; Pan et al. 2022; Akdel et al. 2022).

Towards characterising variant effects, the Critical Assessment of Genome Interpretation (CAGI) challenge invites different research groups to blindly predict the effect of various experimentally characterised mutations. Within the sixth edition of this challenge (CAGI6), thirteen tasks were released, ranging from variation in specific proteins like Calmodulin, to annotation of mutations for clinical classification, and annotation of all missense mutations across the human genome. In this work, we present the findings observed through participation in gene-specific CAGI6 challenges addressing the effect of mutations on Calmodulin (CaM) thermostability, and similarly the effect of mutations on Mitogen-activated protein kinases 1 and 3 (MAPK1 and MAPK3) change in Gibb’s free energy of folding (ΔΔ*G*). We have undertaken these challenges by generating 5 predictions from our tools (SDM, mCSM, DUET, DynaMut and DynaMut2), and one additional normal model analysis-based tool (ENCoM), we have previously utilised in our mutation analyses.

**Fig. 1:**
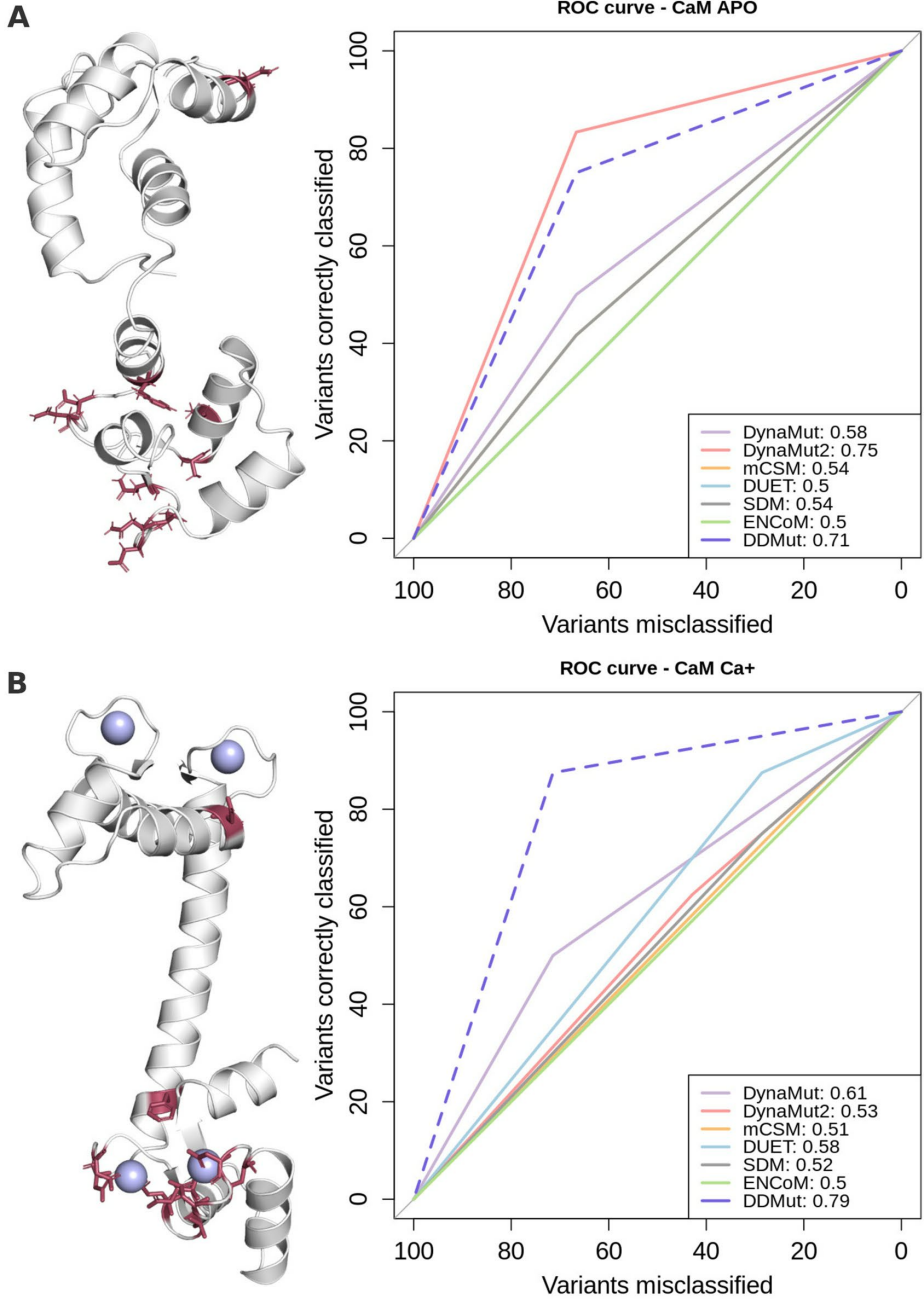
Performance evaluation of classification task for mutations on the Calmodulin challenge. Panel **A** shows the distribution of mutations on 3D structures for the Calmodulin in APO form on the left and ROC plot with performance for each of the 7 methods assessed in this study on the right. Similarly, panel B depicts the structure of Calmodulin bound to Ca^2+^ (purple spheres) on the left and ROC plot on the right side. In both panels, structures are coloured in grey and represented as cartoons, while mutations are coloured in red and depicted as sticks. The ROC curve for DDMut is shown as dashed lines since this method was developed after the challenge (color figure online)

MAPK1 (ERK2) and MAPK3 (ERK1) are serine/threonine kinases, that are active in the Ras-Raf-MEK-ERK signal transduction pathway and regulate a series of vital cellular processes, including cell proliferation, transcription, differentiation and cell cycle progression (Lavoie et al. 2020). MAPK1 is activated by phosphorylation strictly carried out by MEK1/2 on Thr185 and Tyr187 residues, while MAPK3 is phosphorylated on Thr202 and Tyr204 (Roskoski 2012). In addition, both proteins can also act as transcriptional repressors despite their kinase activity. CaM is a calcium (Ca^2+^) sensor protein interacting with a range of molecular partners and regulating a variety of biological processes. The two domains of CaM adopt different conformations in the absence and presence of Ca^2+^ (Fig. 1) being ideal for detecting and responding to a diverse number of intracellular concentrations of Ca^2+^ (Chin and Means 2000). Missense mutations in the genes encoding CaM have been previously shown to be related to ventricular tachycardia and sudden cardiac death (Nyegaard et al. 2012).

Collectively, we have predicted the thermodynamic stability effect of mutations across the three genes, using a range of predictors on both *holo*- and *apo*-forms of the respective crystallised structures. Our findings suggest that mutations observed within 5 Å of important functional sites, specifically ATP-binding and phosphorylation sites within MAPK1 and MAPK3, and Ca^2+^ binding sites within CaM, likely conferred both destabilisation and conformational effects.

## Results and discussion

### MAPK1 protein stability changes highlight possible effects on phosphorylation and catalysis

When considering effects of mutations within MAPK1, predictions on both the inhibitor-bound (1TVO; Table S1; Fig. S1) and phosphorylated ATP-bound (5V60; Table S2; Fig. S2) structures highlighted mutations L121I, R135K, R191H, L200F as highly destabilising, observed across more than one predictor. Of these, R191H localises within 5 Å of the phosphorylation sites, Thr185 and Tyr187 (Fig. S4). Considering the associated moderate decrease in entropy (Δ*S*) observed upon this mutation, the observed destabilisation potentially confers local rigidity, which may be detrimental to protein function by directly affecting phosphorylation. In contrast, based on our computational methods, none of the highly destabilising mutations tested localised within 5 Å of ATP binding, while only E33Q, which conferred negligible to moderate reductions in stability (Tables S1, S2), localised within interaction distance of this ligand. Considering the proximity of E33Q to ATP (5V60) binding, it is possible that E33Q may impart mild effects on the catalytic function of MAPK1. Interestingly, experimental results show that most mutations exhibit neutral/small changes in stability for both structures, except for R191H on the inhibitor-bound structure, which shows a change in stability around 1 kcal/mol (Figs. S1, S2).

Overall, for mutations within the inhibitor-bound structure of MAPK1, ENCoM and SDM had the best and worst performance of the tested methods in predicting ΔΔ*G*, with a Pearson correlation of 0.79 and 0.03, respectively (Table 1). Interestingly, when testing out DDMut (Fig. S3), our most recent approach in predicting ΔΔ*G* upon missense mutations using deep learning, we showed a Pearson correlation of 0.43, which outperformed our previous methods used in the CAGI competition.

**Table 1:** Performance evaluation for regression task for mutations on the MAPK1 and MAPK3 challenges

Method	Method	Pearson	Kendall	Spearman	RMSE
MAPK1 inhibitor-bound (1TVO)	DynaMut	−0.17	−0.09	−0.12	0.74
	DynaMut2	−0.34	−0.30	−0.36	1.03
	mCSM	−0.52	−0.36	−0.41	1.2
	DUET	−0.44	−0.32	0.40	1.16
	SDM	0.03	−0.06	−0.1	0.94
	ENCoM	0.79	0.21	0.32	0.33
	DDMut	0.43	0.32	0.26	0.60
MAPK1 phosphorylated (5V60)	DynaMut	0.03	−0.04	−0.02	0.76
	DynaMut2	−0.49	−0.34	−0.42	0.83
	mCSM	−0.35	−0.19	−0.27	0.93
	DUET	−0.43	−0.31	−0.36	1.05
	SDM	−0.48	−0.31	−0.42	1.05
	ENCoM	0.19	0.08	0.13	0.41
	DDMut	0.03	0.06	0.11	0.56
MAPK3 inhibitor-bound (4QTB)	DynaMut	0.05	−0.02	0.09	2.64
	DynaMut2	−0.21	−0.17	−0.26	3.11
	mCSM	−0.17	−0.29	−0.36	3.20
	DUET	−0.14	−0.17	−0.23	3.16
	SDM	−0.19	−0.23	−0.27	3.23
	ENCoM	0.30	0.08	0.15	2.52
	DDMut	−0.20	−0.05	−0.06	3.13
MAPK3 phosphorylated (2ZOQ)	DynaMut	0.57	0.47	0.57	1.42
	DynaMut2	0.62	0.38	0.50	1.17
	mCSM	0.71	0.53	0.68	1.10
	DUET	0.74	0.66	0.82	1.01
	SDM	0.49	0.26	0.44	1.38
	ENCoM	0.39	0.37	0.45	1.53
	DDMut	−0.38	−0.26	−0.35	1.68

Predictions are assessed for 7 different predictors (DynaMut, DynaMut2, mCSM, DUET, SDM, ENCoM and DDMut) on inhibitor-bound and phosphorylated structures of MAPK1 and MAPK3. Performance is assessed in terms of the correlation coefficients Pearson, Kendall and Spearman, and Root Mean Squared Error (RMSE)

Conversely, D235V and E322V were observed to confer stabilisation to both MAPK1 structures. Of these, the corresponding entropic effects suggest opposite effects on local conformation. Specifically, D235V-conferred stabilisation is associated with a mild increase in entropy, suggesting the mutation also imparts local flexibility, while E322V is associated with a mild decrease in entropy, suggesting local rigidity. Neither of these mutations is within interaction distance (5 Å) of either ligand or phosphorylation sites, suggesting that these changes in conformation may not directly interfere with these MAPK1 functions, but rather through downstream effects. These are corroborated by the experimental results which show little to no change in free energy of folding for all mutations, especially the phosphorylated structure (Fig. S2).

When predicting the effects of mutations on the phosphorylated structure of MAPK1, again ENCoM shows the best performance with a Pearson correlation of 0.19. Surprisingly, DynaMut2, DUET and SDM showed a negative correlation varying from −0.35 to −0.49, and predicting more drastic changes in protein stability for most variants (Table 1). Importantly, despite these negative correlations, all methods performed well at correctly identifying mutations as either stabilising or destabilising.

Further to our initial analyses, a performance evaluation of our methods was also carried out to assess their ability to distinguish between stabilising and destabilising mutations (classification by regression). Performances for the inhibitor-bound MAPK1 structure ranged from AUC values from 0.41 to 0.62, from SDM and ENCoM, respectively. On the phosphorylated MAPK1, DynaMut performed well, with an AUC of 0.75, and ENCoM had an AUC of 1 (Table S3).

Considering these different analyses, overall trends show that ENCoM, across both inhibitor-bound *holo*- and phosphorylated *apo*-forms best describes the stability changes imparted in MAPK1 upon mutation. This is likely because kinases like MAPK1 are more subject and sensitive to conformational changes, which are accounted for within the ENCoM method through harmonic motion calculations. Beyond ENCoM, our newer method, DDMut, offered the second-best performance on MAPK1 mutant stability predictions, which also accounts for conformational fluctuations via torsion angles. The other methods tested lack this consideration, while also using simpler machine learning frameworks compared to a more advanced deep-neural network, used by DDMut.

### MAPK3 protein stability changes highlight possible effects on phosphorylation and catalysis

Of the mutations analysed for MAPK3 (Fig. S5), we observed that I73M, A160T, E214D and V290A were observed destabilising across both the inhibitor-bound (4QTB; Table S4; Fig. S6) and the phosphorylated (2ZOQ; Table S5; Fig. S7) structures, in congruence with the experimental results on the phosphorylated structure. While neither structure was bound to ATP, mutation I73M mapped at the ATP binding site, where the large destabilisation effect observed could directly impact on MAPK3 catalytic activity. Experimental results confirm this mutation as greatly impacting both structures but with a more drastic effect for the inhibitor-bound structure. By corollary, this mutation also localised within 5 Å of the inhibitor binding site, while none of the other mutations predicted as highly destabilising were observed to do so. Notably, of the analysed mutations, T198I localises within 5 Å of the phosphorylation sites Thr202 and Tyr204. This mutation was observed to highly stabilise the phosphorylated structure (2ZOQ; Table S5) across virtually all predictors, which was also corroborated by the experimental results. Furthermore, considering its observed associated decrease in entropy, this mutation may also lead to local conformational changes through rigidification.

Finally, mutations P336Q and E339V were also observed to confer protein stabilisation, of which, P336Q, particularly when analysed within the phosphorylated structure (2ZOQ; Table S5), was associated with decreased entropy, conferring local rigidification. Both of the stabilising mutations, however, mapped far away from ligand or phosphorylation sites, suggesting that these changes in conformation may not directly lead to functional deleteriousness, but confer possible downstream effects. Experimental results confirmed these mutations as stabilising as shown in Fig. S6.

Overall, ENCoM showed the best performance across all methods for predicting the effects of mutations on the inhibitor-bound structure (4QTB), achieving a Pearson correlation of 0.30 (Table S4). For the phosphorylated structure (2ZOQ), DUET, mCSM and DynaMut2 are the top-performing methods, achieving Pearson correlations of 0.74, 0.71 and 0.62, respectively (Table 1). Similarly to the classification by regression analysis on the 3D structures of MAPK1, the performance of methods ranged AUC values from 0.35 to 0.65 for DynaMut2 and DDMut (Table S6), respectively, on the inhibitor-bound structure of MAPK3. For mutations on the phosphorylated structure of MAPK3, DynaMut and DUET achieved the top performance with AUC values of 0.75 and 0.81, respectively.

Considering our analyses in combination, it is again observed that ENCoM is best suited to estimate the stability changes of mutations within the inhibitor-bound MAPK3. However, our other tools DynaMut, DynaMut2, mCSM, DUET and SDM were more suited to predict mutations within the phosphorylated MAPK3. These differences in performance across the *holo*- and phosphorylated *apo*-forms suggest that phosphorylation may improve the local stability of the protein and that this stability is strong in MAPK3 compared to its tested homolog MAPK1. This can be exemplified in our metrics as our higher-performing methods better account for the mutant local environment within their predictions, compared to ENCoM and DDMut (Fig. S8), which are better representative of conformational changes, observed in the inhibitor-bound holo-MAPK3.

### CaM protein stability changes possibly effect Ca^2+^ binding and subsequent function

When considering resultant ΔΔ*G* values obtained for the CaM mutations, it was observed that mutations F89L and E140G consistently led to large reductions in stability across both Ca^2+^ bound (1CLL) and unbound structures (1DMO; Fig. S9; Tables S7, S8). Of these, E140G localises within the interaction distance (5 Å) of Ca^2+^ ions. When comparing values for E140G across structures, it was observed that the extent of destabilisation was lower for the Ca^2+^ bound state (1CLL), suggesting that Ca^2+^ ions help to confer local stability. Notably, however, E140G destabilisation within the Ca^2+^ bound state was accompanied by a large increase in entropy, suggesting that this mutation, which leads to a loss of negatively charged Glu to a flexible, more neutral Gly, also leads to increased local flexibility. This local flexibility may disrupt Ca^2+^ binding, thereby being deleterious to CaM function.

Further to that, mutation E104A, occurring at the same position, was observed to confer moderate destabilisation effects within the Ca^2+^ bound structure (1CLL), compared to the unbound structure. This was also accompanied by a moderate increase in local entropy, suggesting that, within the Ca^2+^ bound state, this mutation, again resulting in a loss of negative Glu to neutral Ala, leads to an increase in local flexibility. Similarly to E140G, this mutation is thought to be deleterious to CaM function, although to a lower extent, possibly due to a lower flexibility of mutant Ala compared to mutant Gly.

In terms of performance with the experimental results, here DynaMut2 outperformed all other methods achieving a ROC AUC of 0.75 for mutations on the CaM APO structure (1DMO), followed by DynaMut1 and mCSM with 0.58 and 0.54 AUC, respectively (Fig. 1A). When analysing the same set of mutations on the phosphorylated structure of CaM (1CLL), DynaMut1 and DUET showed the best performance across all methods achieving AUC values of 0.61 and 0.58, respectively, and were closely followed by DynaMut2 with an AUC of 0.53 (Fig. 1B). These results have been generated after removal of mutation E140G which has been excluded from the challenge by the assessors.

Finally, we have additionally evaluated the predictive performance of our most recent deep learning approach, DDMut (Tables S7, S8), for variants on both protein structures. DDMut showed a consistent performance, achieving comparable performance with DynaMut2 on variants for CaM in the APO form (AUCs of 0.71), and outperforming all other methods on variants on CaM bound to Ca^2+^ with AUC of 0.79.

## Conclusion

Our research has demonstrated the significance of incorporating dynamic aspects of proteins in the computational prediction of the effects of mutations. By considering the intricate motions and interactions within proteins, methods encompassing these dynamics, such as ENCoM, DynaMut and DynaMut2, have exhibited superior performance overall. Additionally, the emergence of novel deep learning approaches, including DDMut, has shown great promise in this field. As highlighted in the MAPK1 vs MAPK3 analyses, we have observed that the tested methods have optimised applications depending on protein conformational nature. Specifically, methods like ENCoM and DDMut are optimised towards proteins subject to conformational changes, while our other methods better account for local environmental changes upon mutation. Finally, as we further advance in the field of computational prediction, it will become increasingly crucial to recognize and analyse arising trends through the application of these methods. In doing so, we can better understand the underlying principles governing mutational effects and propel our understanding of biological systems forward, towards precision medicine efforts.

## Methods

### MAPK1 and MAPK3 challenges

For these challenges, eleven and twelve missense variants were selected from the COSMIC database (Tate et al. 2019) for MAPK1 and MAPK3, respectively. These were experimentally assessed by circular dichroism (CD) and intrinsic fluorescence spectra to determine thermodynamic stability at different concentrations of denaturant. These measurements were used to calculate changes in the Gibbs Free Energy of Folding (ΔΔ*G*) values for MAPK1 and MAPK3, both in phosphorylated and unphosphorylated forms. The CAGI challenge sought to predict these ΔΔ*G* values and the catalytic efficiency upon missense mutations, as determined by the fluorescence assays of the phosphorylated forms for each protein variant. Experimental ΔΔ*G* values provided by the assessors of these two challenges were directly used to estimate performance metrics for all predictive methods discussed in this study.

Experimental structures for MAPK1 and MAPK3 were derived from RCSB PDB as suggested by the data providers. For MAPK1, entries 1TVO and 5V60 were used and represent the protein bound to a small molecule inhibitor and the phosphorylated protein, respectively (Fig. S4). For MAPK3, entries 4QTB and 2ZOQ were used, representing this protein in complex with a small molecule inhibitor and the phosphorylated protein, respectively (Fig. S5).

Each variant was then mapped onto experimental structures using the Uniprot accession codes P28482 and P27361 for MAPK1 and MAPK3, respectively, using PDBSWS (Martin 2005).

### Calmodulin challenge

A library of 16 point mutations was assessed via CD by measuring melting temperature (*T*_m_) and percentage of unfolding upon thermal denaturation. The ultimate goal being the prediction of (1) the *T*_m_ and percentage of unfolding values for isolated CaM variants under Ca^2+^-saturating conditions and in the APO form; and (2) whether the point mutation stabilises or destabilises the protein.

Mutated positions were provided according to the human CaM (Uniprot accession P0DP23). Variants were then mapped on the experimental structures for CaM bound to Ca^2+^ (1CLL) and in the APO form (1DMO) available in the PDB (Fig. 1), using the PDBSWS mapping.

While the goal of this challenge was to predict *T*_m_, we considered this to be equivalent to ΔΔ*G*, and submitted our entries with the predicted ΔΔ*G* values from our methods with no transformation. Across all our methods, a positive value denoted stabilisation, while a negative value denoted destabilisation. For each mutation, the ground truth labels, stabilise and destabilise, were defined based on the experimental percentage of unfold provided by the assessors of this challenge. Entries with a percentage of unfold smaller than the wild-type reference were considered to be stabilising and destabilising otherwise.

### Structure-based machine learning approaches to predict changes in protein stability

For all 3 challenges described, our team submitted 6 different predictions: 5 *in-house* approaches (SDM, mCSM, DUET, DynaMut and DynaMut2), the majority of which leverage physicochemical properties and distance pattern signatures extracted from protein structure data (Pandurangan et al. 2017; Pires et al. 2014a, 2014b; Rodrigues et al. 2018, 2021; Zhou et al. 2023); and ENCoM (Frappier et al. 2015), a normal mode analysis (NMA) approach incorporated in our mutational analysis pipelines. Each mutation and PDB structure were input into our webservers and results were compiled accordingly. Our methods predict the effects of mutations in terms of changes in the Gibbs Free Energy of folding, ΔΔ*G*, which can be appropriately compared with the actual ΔΔ*G* values for MAPK1 and MAPK3 challenges, and were also used as a direct measure of melting temperature values and the classification task (whether a variant stabilises or destabilises the protein) from the CaM challenge. A brief description of each method is available in Table S9.

The effects of these mutations on their respective protein structures were analysed collectively as previously reported (Portelli et al. 2018, 2020a, b, 2021, 2023b). Briefly, we considered ΔΔ*G* values |*x*|< 0.05 as negligible, 0.05 <|*x*|< 0.5 as mild, 0.5 <|*x*|< 1.0 as moderate, and |*x*|> 1.0 as large. Notably, while a decrease in stability is more commonly associated with deleterious effects on protein function, an increase in stability may also confer deleteriousness (Stefl et al. 2013), due to changes in local conformation via rigidification (Tokuriki and Tawfik 2009). To illustrate the effects of mutations on protein conformation, and further aid in mutation characterisation we also report protein entropy, Δ*S*, as calculated by ENCoM.

## Supplementary Information

Below is the link to the electronic supplementary material.

Supplementary file1 (DOCX 3884 KB)

## Data Availability

The dataset of mutations and protein structures used in this study are available at https://bitbucket.com/ascherlab/cagi6.
