# Supplementary material for: Exploring the effects of missense mutations on protein thermodynamics through structure-based approaches: findings from the CAGI6 challenges
Source: Hum Genet. 2024 Jan 16;144(2-3):327–35. doi: 10.1007/s00439-023-02623-4 (PMC11976750; doi:10.1007/s00439-023-02623-4)
Supplement: Supplementary file 1 — Supplementary file1 (DOCX 3884 KB) [file 439_2023_2623_MOESM1_ESM.docx]

**SUPPLEMENTARY MATERIAL**

**Exploring the effects of missense mutations on protein thermodynamics through structure-based approaches: Findings from the CAGI6 challenges**

Carlos H. M. Rodrigues^1,2^, Stephanie Portelli^1,2^, David B. Ascher^1,2^

1 Computational Biology and Clinical Informatics, Baker Heart and Diabetes Institute, Melbourne, VIC 3004, Australia

2 School of Chemistry and Molecular Biosciences, University of Queensland, St Lucia, QLD 4072, Australia

Correspondence: d.ascher@uq.edu.au; Tel.: +61 7 336 53991 (D.B.A)

# FIGURES


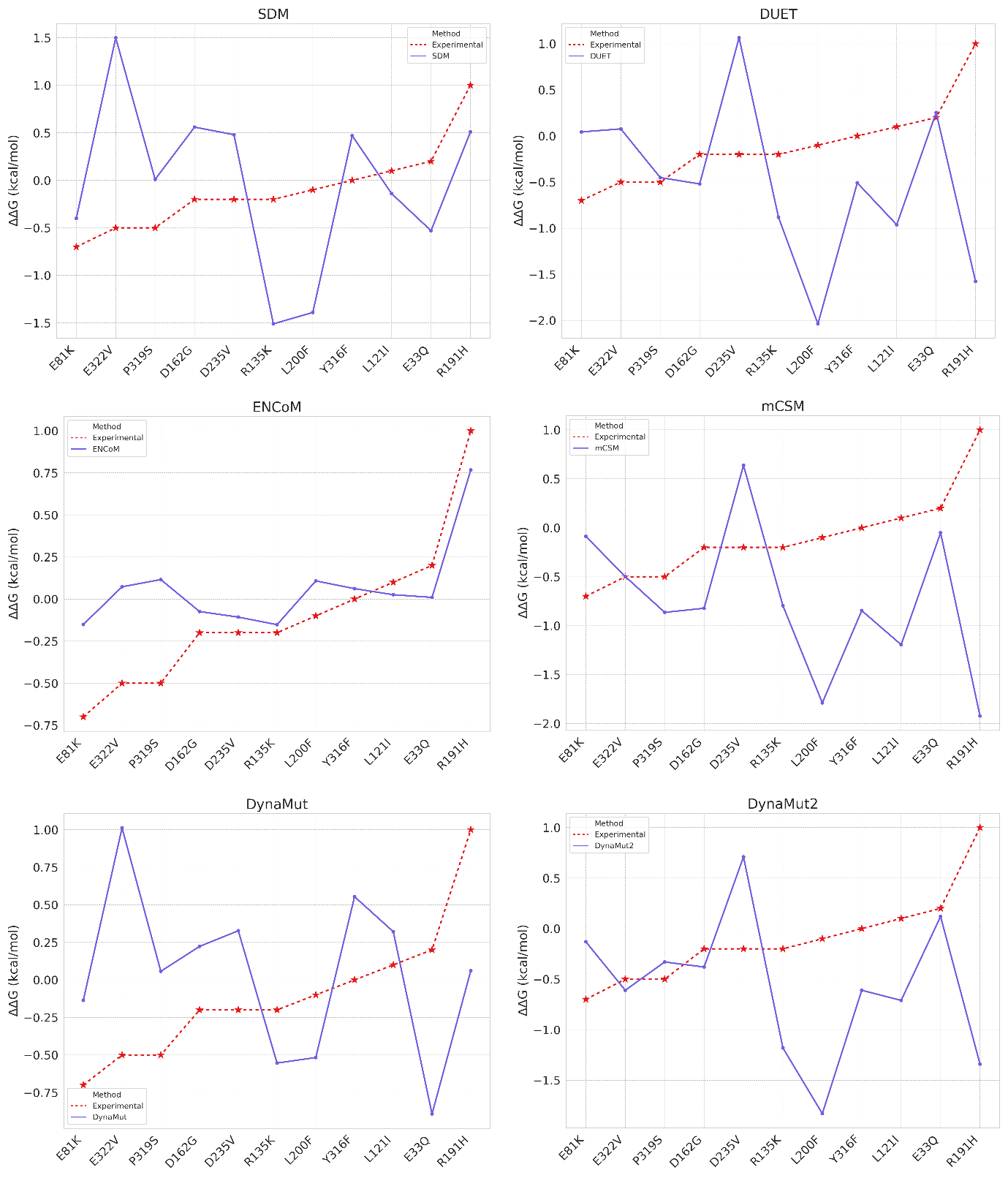


**Figure S1** - ΔΔG prediction for all 6 predictors submitted to the MAPK1 challenge on the structure of MAPK1 bound to an inhibitor. Each panel shows the output predictions for a single computational method shown as solid lines and ground truth experimental information as red stars and dashed lines.


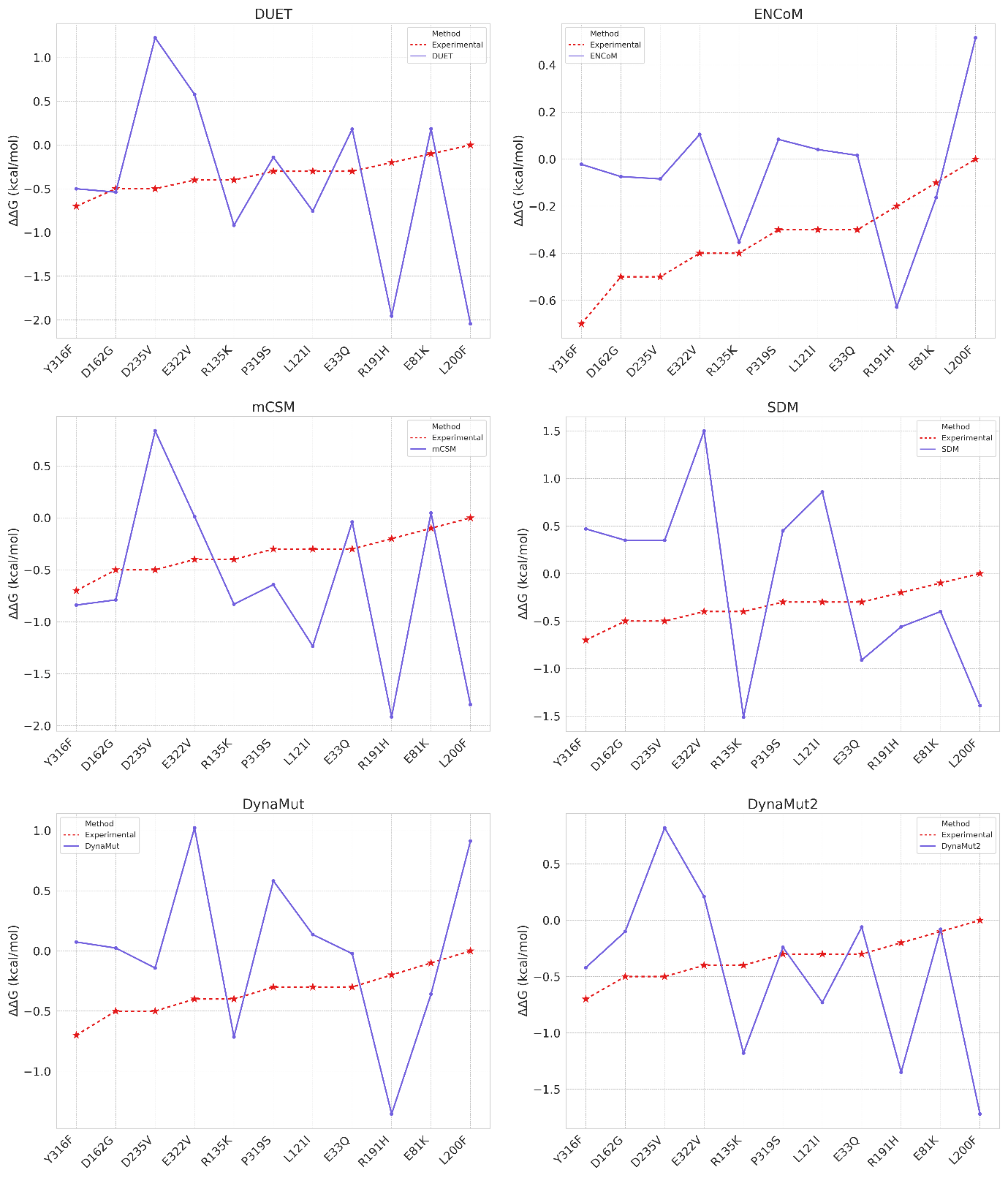


**Figure S2** - ΔΔG prediction for all 6 predictors submitted to the MAPK1 challenge on the phosphorylated structure of MAPK1. Each panel shows the output predictions for a single computational method shown as solid lines and ground truth experimental information as red stars and dashed lines.


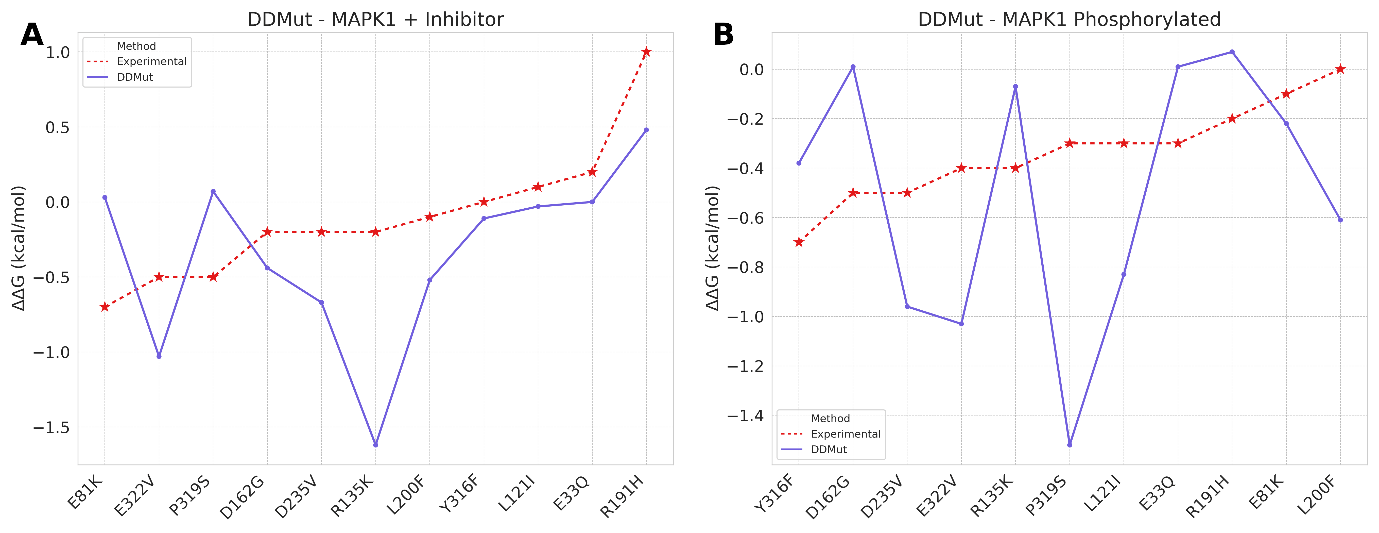


**Figure S3** - ΔΔG prediction DDMut on the MAPK1 challenge. A) shows a comparison between DDMut predictions and experimental values of ΔΔG on the MAPK1 bound to an inhibitor, while B) summarises the comparison of results using the phosphorylated structure of MAPK1.


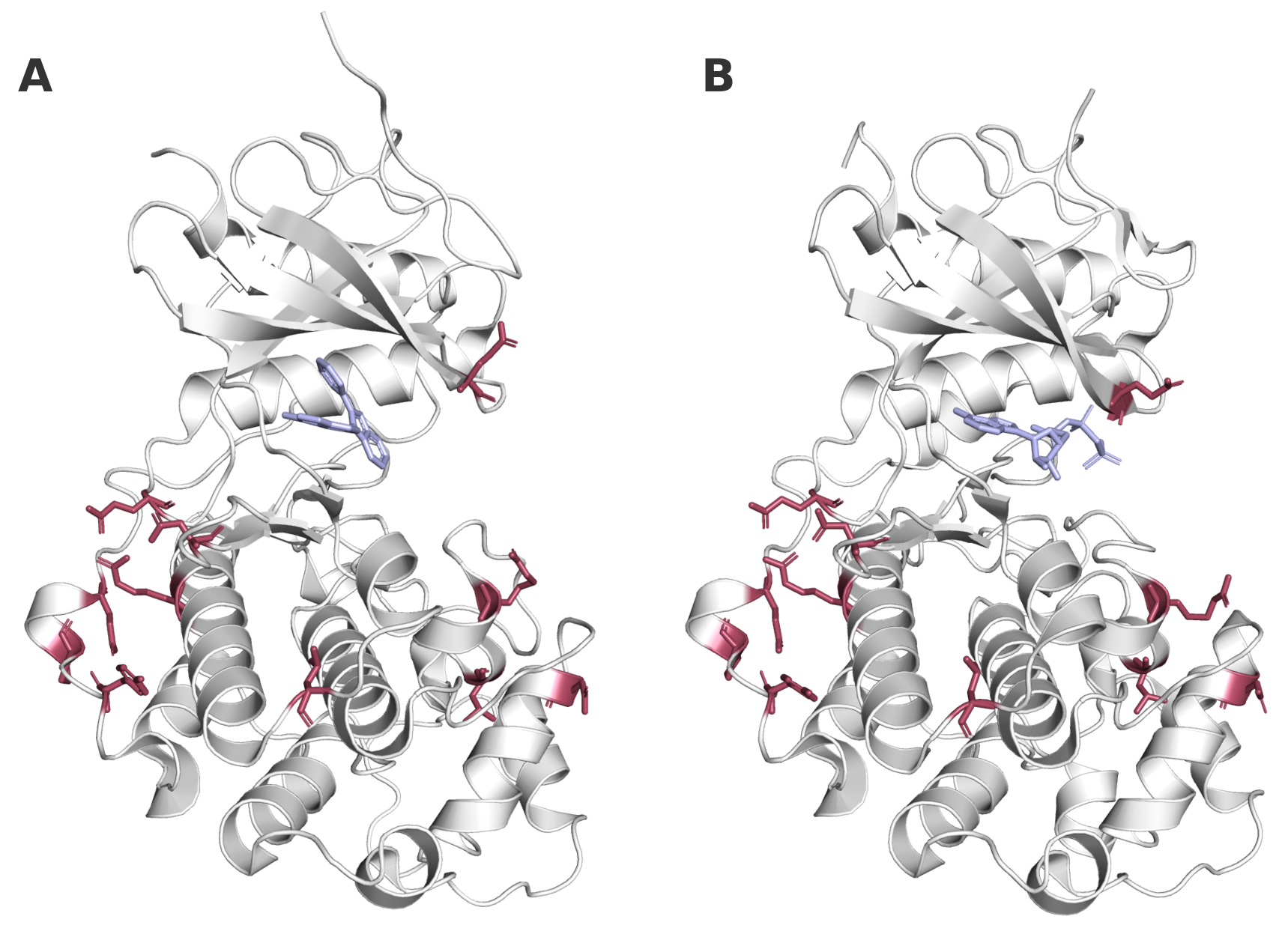


**Figure S4** - Variants mapped to experimental structures of MAPK1 protein. A) shows the structure of MAPK1 bound to an inhibitor (purple) and B) displays the structure of MAPK1 bound to an ATP-like molecule (purple). All variants have been highlighted in stick representation and coloured in red in both structures.





**Figure S5** - Variants mapped to experimental structures of MAPK3 protein. A) shows the structure of MAPK3 bound to an inhibitor (purple) and B) displays the structure of phosphorylated MAPK3 bound to a small-molecule (purple). All variants have been highlighted in stick representation and coloured in red in both structures.


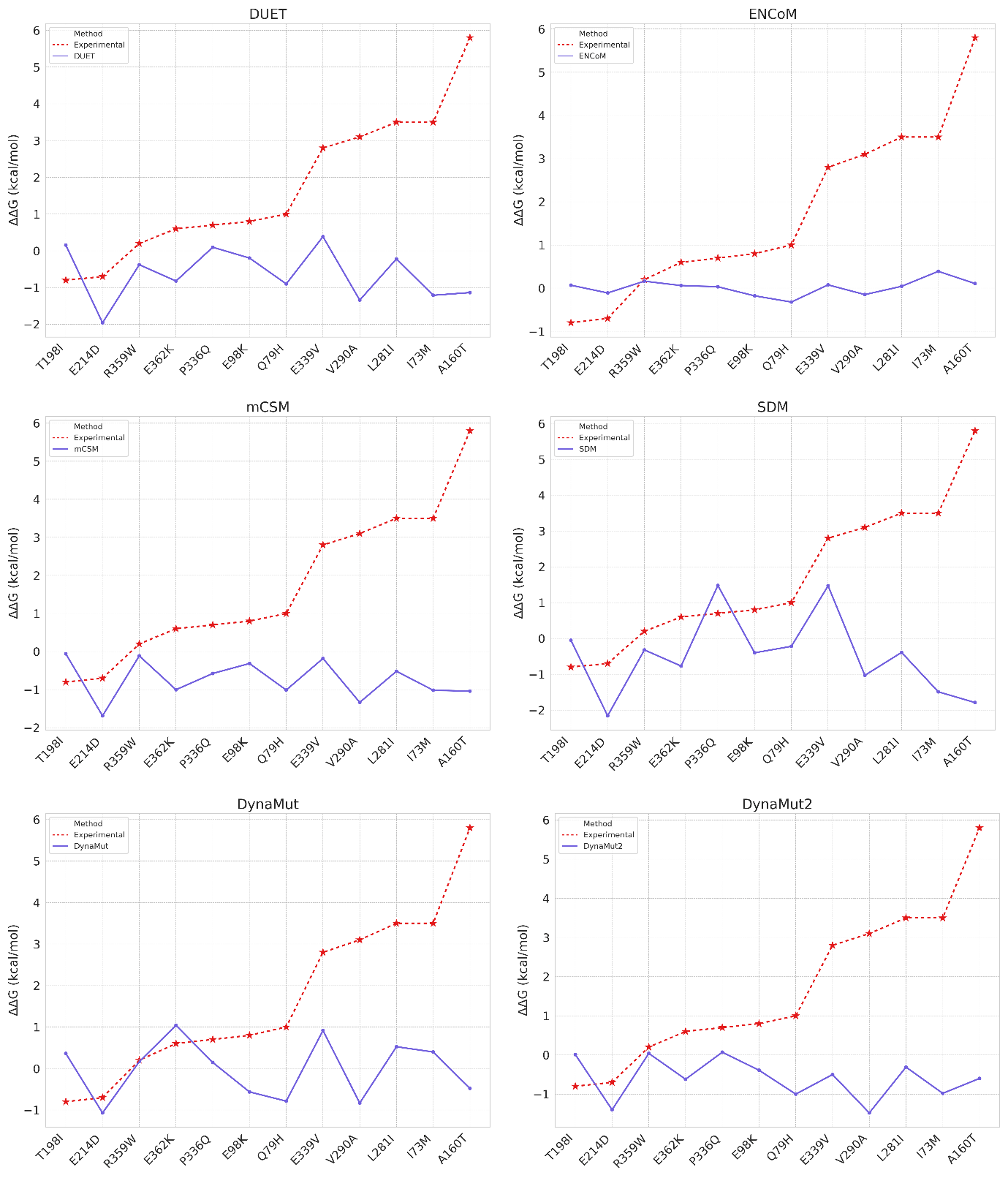


**Figure S6** - ΔΔG prediction for all 6 predictors submitted to the MAPK3 challenge on the structure of MAPK3 bound to an inhibitor. Each panel shows the output predictions for a single computational method shown as solid lines and ground truth experimental information as red stars and dashed lines.


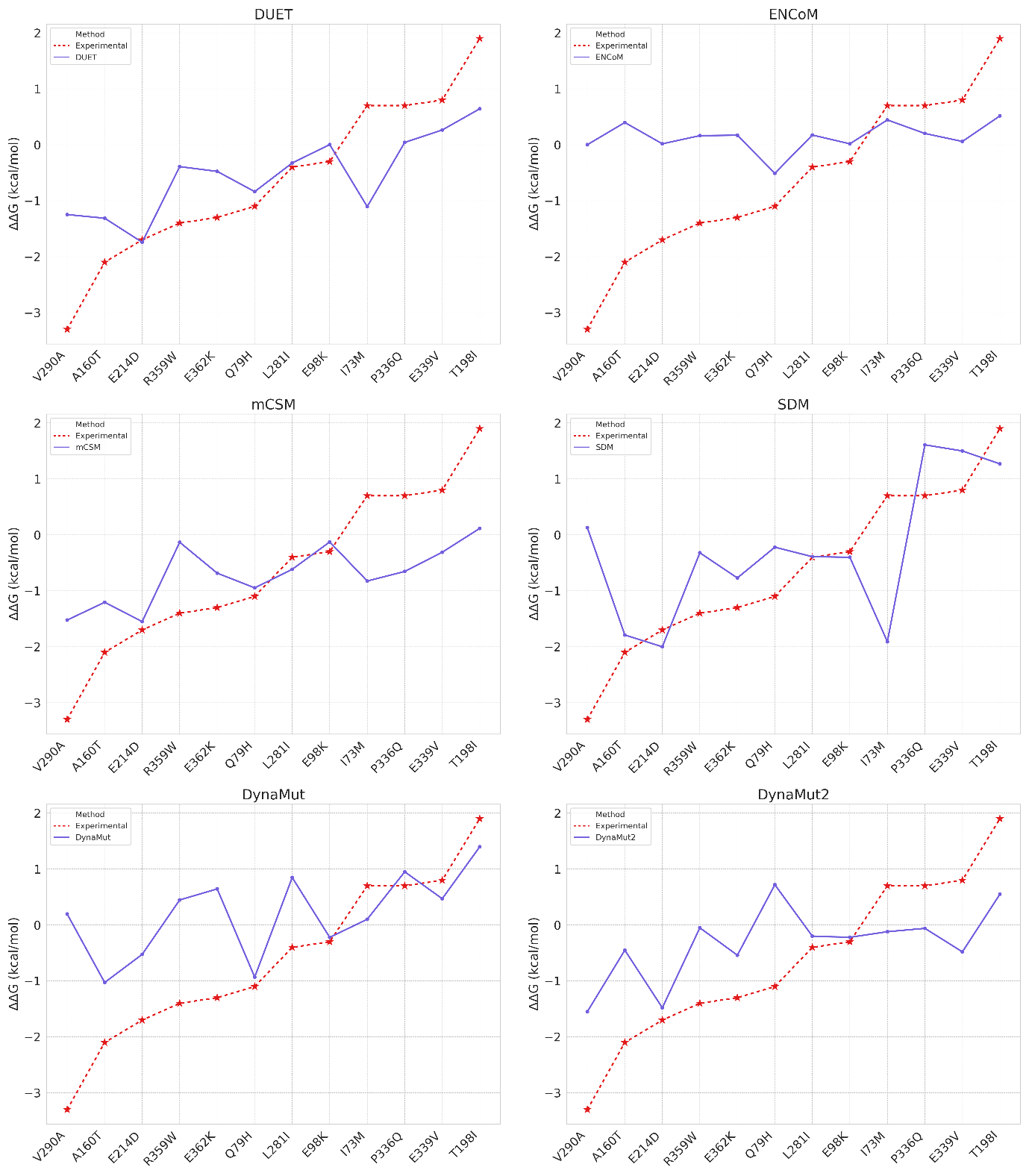


**Figure S7** - ΔΔG prediction for all 6 predictors submitted to the MAPK3 challenge on the phosphorylated structure of MAPK3. Each panel shows the output predictions for a single computational method shown as solid lines and ground truth experimental information as red stars and dashed lines.


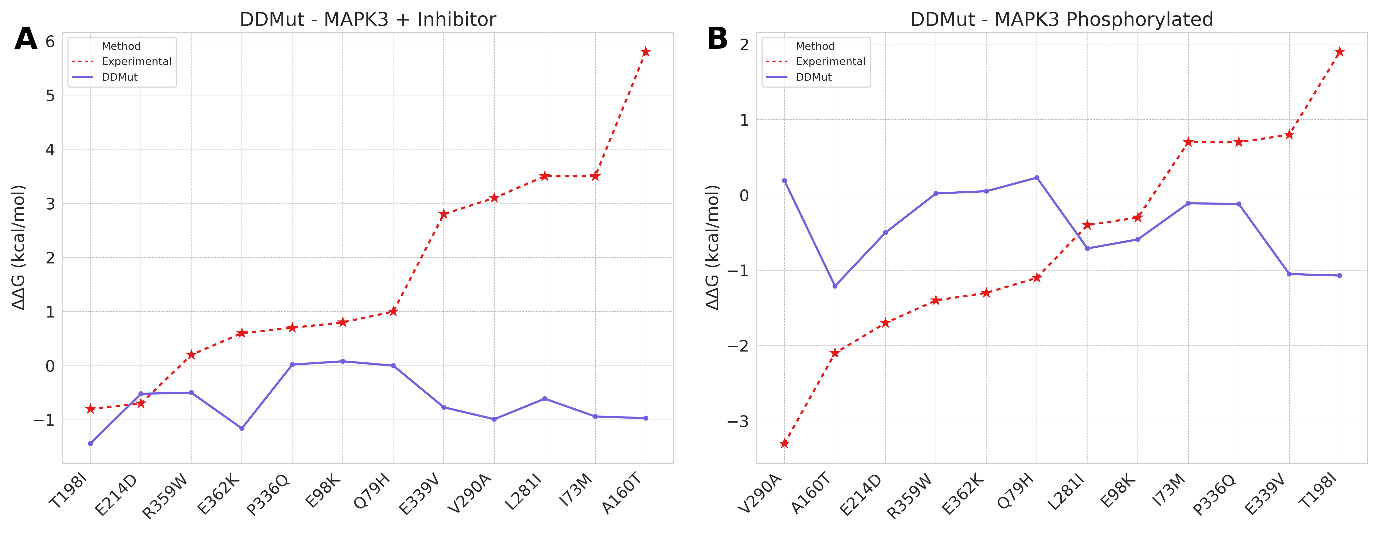


**Figure S8** - ΔΔG prediction DDMut on the MAPK3 challenge. A) shows a comparison between DDMut predictions and experimental values of ΔΔG on the MAPK3 bound to an inhibitor, while B) summarises the comparison of results using the phosphorylated structure of MAPK3.


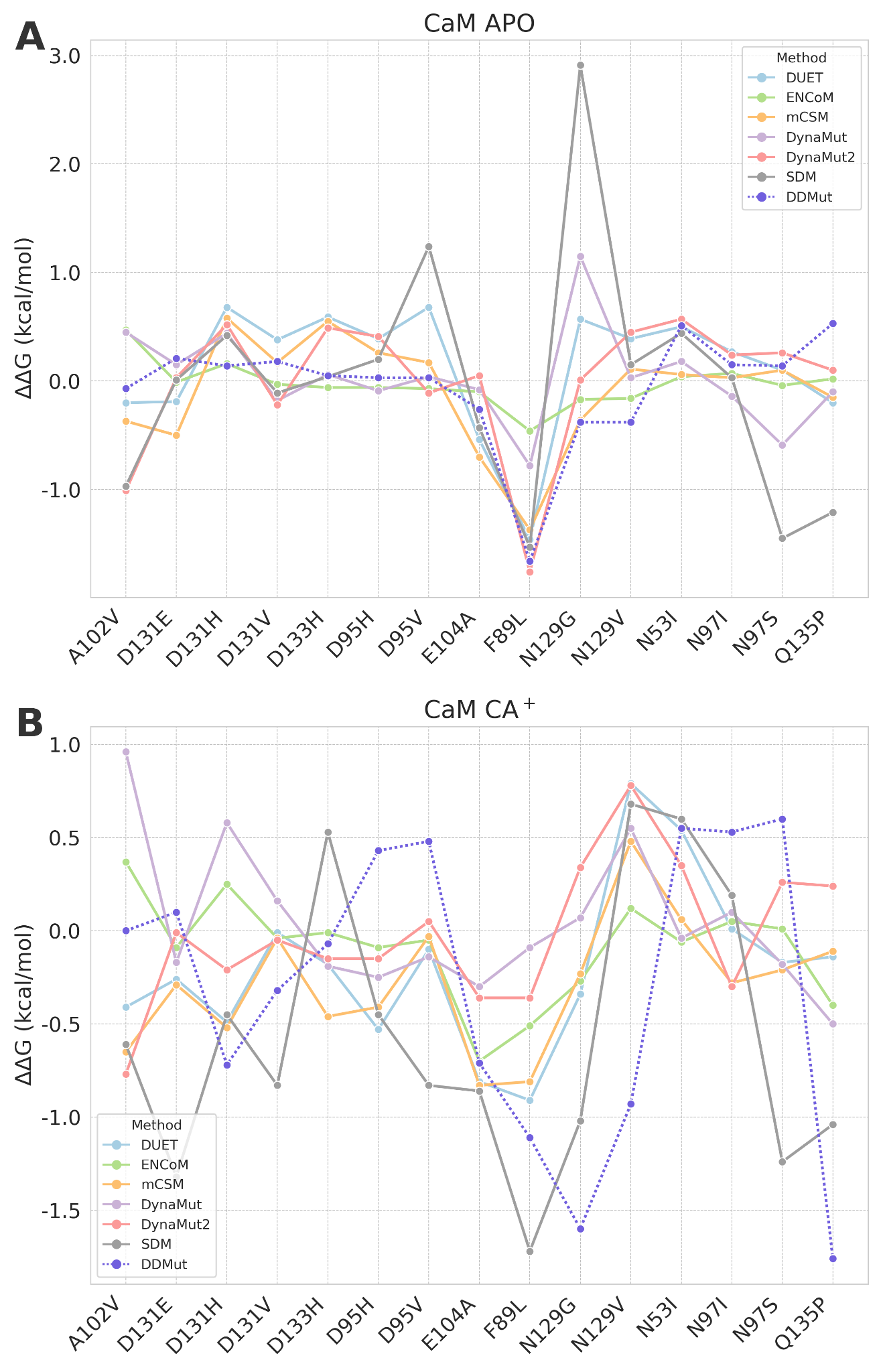


**Figure S9** - ΔΔG prediction for all 6 predictors submitted to the Calmodulin challenge and DDMut predictions. Results for DDMut are shown as dashed lines since this method was developed after the challenge.

# TABLES

**Table S1** - Predicted effects of mutations on protein stability and flexibility for the MAPK1 protein bound to an inhibitor (PDB: 1TVO).

| **Mutation** | **Experimental ΔΔG** | **Distance to inhibitor (Å)** | **ΔS ENCoM** | **DUET** | **ENCoM** | **mCSM** | **SDM** | **DynaMut** | **DynaMut2** | **DDMut** |
| --- | --- | --- | --- | --- | --- | --- | --- | --- | --- | --- |
| R135K | -0.200 | 16.700 | 0.19 | -0.881 | -0.152 | -0.796 | -1.51 | -0.554 | -1.18 | -1.62 |
| E33Q | 0.200 | 8.149 | -0.013 | 0.254 | 0.01 | -0.051 | -0.53 | -0.893 | 0.12 | 0.00 |
| D235V | -0.200 | 23.276 | 0.134 | 1.065 | -0.107 | 0.638 | 0.48 | 0.327 | 0.71 | -0.67 |
| R191H | 1.000 | 12.288 | -0.959 | -1.577 | 0.767 | -1.922 | 0.51 | 0.061 | -1.34 | 0.48 |
| L121I | 0.100 | 16.729 | -0.032 | -0.963 | 0.026 | -1.194 | -0.14 | 0.321 | -0.71 | -0.03 |
| L200F | -0.100 | 25.127 | -0.135 | -2.037 | 0.108 | -1.788 | -1.39 | -0.517 | -1.83 | -0.52 |
| P319S | -0.500 | 25.624 | -0.144 | -0.451 | 0.116 | -0.864 | 0.01 | 0.057 | -0.33 | 0.07 |
| E81K | -0.700 | 11.056 | 0.189 | 0.044 | -0.151 | -0.088 | -0.4 | -0.136 | -0.13 | 0.03 |
| D162G | -0.200 | 9.565 | 0.093 | -0.52 | -0.074 | -0.822 | 0.56 | 0.223 | -0.38 | -0.44 |
| E322V | -0.500 | 21.927 | -0.092 | 0.077 | 0.073 | -0.498 | 1.5 | 1.012 | -0.61 | -1.03 |
| Y316F | 0.000 | 21.794 | -0.077 | -0.508 | 0.062 | -0.846 | 0.47 | 0.554 | -0.61 | -0.11 |

**Table S2** – Predicted effects of mutations on protein stability and flexibility for the phosphorylated MAPK1 protein bound to an ATP-like molecule (PDB: 5V60).

| **Mutation** | **Experimental ΔΔG** | **Distance to ligand (Å)** | **ΔS ENCoM** | **DUET** | **ENCoM** | **mCSM** | **SDM** | **DynaMut** | **DynaMut2** | **DDMut** |
| --- | --- | --- | --- | --- | --- | --- | --- | --- | --- | --- |
| P319S | -0.300 | 26.480 | -0.104 | -0.14 | 0.084 | -0.642 | 0.45 | 0.582 | -0.24 | -1.52 |
| R135K | -0.400 | 16.918 | 0.441 | -0.918 | -0.353 | -0.831 | -1.51 | -0.715 | -1.18 | -0.07 |
| L121I | -0.300 | 15.419 | -0.052 | -0.755 | 0.041 | -1.234 | 0.86 | 0.136 | -0.73 | -0.83 |
| L200F | 0.000 | 22.055 | -0.644 | -2.045 | 0.516 | -1.797 | -1.39 | 0.913 | -1.72 | -0.61 |
| E81K | -0.100 | 11.172 | 0.204 | 0.186 | -0.163 | 0.047 | -0.4 | -0.359 | -0.08 | -0.22 |
| Y316F | -0.700 | 22.663 | 0.027 | -0.5 | -0.022 | -0.839 | 0.47 | 0.074 | -0.42 | -0.38 |
| D162G | -0.500 | 10.310 | 0.093 | -0.539 | -0.074 | -0.789 | 0.35 | 0.024 | -0.1 | 0.01 |
| R191H | -0.200 | 10.367 | 0.786 | -1.956 | -0.629 | -1.915 | -0.56 | -1.355 | -1.35 | 0.07 |
| D235V | -0.500 | 20.440 | 0.104 | 1.227 | -0.084 | 0.838 | 0.35 | -0.142 | 0.82 | -0.96 |
| E322V | -0.400 | 22.017 | -0.131 | 0.58 | 0.105 | 0.012 | 1.5 | 1.022 | 0.21 | -1.03 |
| E33Q | -0.300 | 3.573 | -0.019 | 0.182 | 0.016 | -0.039 | -0.91 | -0.023 | -0.06 | 0.01 |

**Table S3** – Performance assessment of classification task prediction of effects of mutations on protein stability and flexibility for different structures of MAPK1.

| **MAPK1 – Inhibitor bound (1TVO)** | | | | | | | |
| --- | --- | --- | --- | --- | --- | --- | --- |
| **Method** | **TN** | **FP** | **FN** | **TP** | **MCC** | **F1** | **AUC** |
| DynaMut | 3 | 1 | 4 | 3 | 0.18 | 0.55 | 0.59 |
| DynaMut2 | 1 | 3 | 1 | 6 | 0.13 | 0.75 | 0.55 |
| mCSM | 0 | 4 | 1 | 6 | -0.24 | 0.71 | 0.43 |
| DUET | 1 | 3 | 3 | 4 | -0.18 | 0.57 | 0.41 |
| SDM | 2 | 2 | 4 | 3 | -0.07 | 0.50 | 0.46 |
| ENCoM | 1 | 3 | 0 | 7 | 0.42 | 0.82 | 0.62 |
| DDMut | 2 | 2 | 2 | 5 | 0.21 | 0.71 | 0.61 |
| **MAPK1 – bound to ATP-like small molecule (5V60)** | | | | | | | |
| **Method** | **TN** | **FP** | **FN** | **TP** | **MCC** | **F1** | **AUC** |
| DynaMut | 1 | 0 | 5 | 5 | 0.29 | 0.67 | 0.75 |
| DynaMut2 | 0 | 1 | 2 | 8 | -0.15 | 0.84 | 0.40 |
| mCSM | 0 | 1 | 3 | 7 | -0.19 | 0.78 | 0.35 |
| DUET | 0 | 1 | 4 | 6 | -0.24 | 0.71 | 0.30 |
| SDM | 0 | 1 | 6 | 4 | -0.35 | 0.53 | 0.2 |
| ENCoM | 1 | 0 | 0 | 10 | 1 | 1 | 1 |
| DDMut | 0 | 1 | 3 | 7 | -0.19 | 0.78 | 0.25 |

**Table S4** - Predicted effects of mutations on protein stability and flexibility for the MAPK3 protein bound to an inhibitor (PDB: 4QTB).

| **Mutation** | **Experimental ΔΔG** | **Distance to inhibitor (Å)** | **ΔS ENCoM** | **DUET** | **ENCoM** | **mCSM** | **SDM** | **DynaMut** | **DynaMut2** | **DDMut** |
| --- | --- | --- | --- | --- | --- | --- | --- | --- | --- | --- |
| R359W | 0.200 | 14.715 | -0.204 | -0.378 | 0.163 | -0.113 | -0.32 | 0.165 | 0.04 | -0.50 |
| E214D | -0.700 | 22.359 | 0.136 | -1.958 | -0.109 | -1.688 | -2.16 | -1.07 | -1.4 | -0.52 |
| I73M | 3.500 | 3.555 | -0.489 | -1.21 | 0.391 | -1.016 | -1.49 | 0.399 | -0.98 | -0.94 |
| E362K | 0.600 | 14.540 | -0.077 | -0.822 | 0.062 | -1.002 | -0.77 | 1.041 | -0.62 | -1.16 |
| E98K | 0.800 | 11.539 | 0.22 | -0.196 | -0.176 | -0.314 | -0.4 | -0.564 | -0.39 | 0.08 |
| A160T | 5.800 | 17.502 | -0.135 | -1.136 | 0.108 | -1.04 | -1.79 | -0.479 | -0.6 | -0.97 |
| V290A | 3.100 | 32.993 | 0.184 | -1.341 | -0.147 | -1.335 | -1.03 | -0.832 | -1.48 | -0.99 |
| L281I | 3.500 | 30.764 | -0.055 | -0.22 | 0.044 | -0.518 | -0.39 | 0.525 | -0.31 | -0.61 |
| Q79H | 1.000 | 8.388 | 0.4 | -0.9 | -0.32 | -1.011 | -0.22 | -0.786 | -1.0 | 0.00 |
| T198I | -0.800 | 21.818 | -0.088 | 0.156 | 0.07 | -0.059 | -0.05 | 0.364 | 0.01 | -1.44 |
| P336Q | 0.700 | 26.457 | -0.042 | 0.097 | 0.034 | -0.576 | 1.48 | 0.146 | 0.07 | 0.02 |
| E339V | 2.800 | 22.119 | -0.098 | 0.385 | 0.078 | -0.181 | 1.47 | 0.917 | -0.5 | -0.77 |

**Table S5** - Predicted effects of mutations on protein stability and flexibility for the phosphorylated MAPK3 protein (PDB: 2ZOQ).

| **Mutation** | **Experimental ΔΔG** | **Distance to ligand (Å)** | **ΔS ENCoM** | **DUET** | **ENCoM** | **mCSM** | **SDM** | **DynaMut** | **DynaMut2** | **DDMut** |
| --- | --- | --- | --- | --- | --- | --- | --- | --- | --- | --- |
| P336Q | 0.700 | 26.717 | -0.252 | 0.042 | 0.202 | -0.654 | 1.61 | 0.951 | -0.06 | -0.12 |
| A160T | -2.100 | 19.138 | -0.497 | -1.313 | 0.398 | -1.205 | -1.79 | -1.028 | -0.45 | -1.21 |
| T198I | 1.900 | 26.614 | -0.645 | 0.643 | 0.516 | 0.116 | 1.27 | 1.398 | 0.55 | -1.07 |
| E214D | -1.700 | 23.255 | -0.021 | -1.743 | 0.017 | -1.55 | -2 | -0.525 | -1.48 | -0.5 |
| E362K | -1.300 | 21.872 | -0.213 | -0.476 | 0.17 | -0.684 | -0.77 | 0.645 | -0.54 | 0.05 |
| I73M | 0.700 | 9.240 | -0.555 | -1.106 | 0.444 | -0.825 | -1.91 | 0.102 | -0.12 | -0.11 |
| E98K | -0.300 | 11.169 | -0.021 | 0.003 | 0.017 | -0.127 | -0.4 | -0.221 | -0.22 | -0.59 |
| L281I | -0.400 | 31.249 | -0.218 | -0.326 | 0.174 | -0.616 | -0.39 | 0.849 | -0.2 | -0.71 |
| V290A | -3.300 | 29.596 | -0.001 | -1.248 | 0.001 | -1.523 | 0.13 | 0.196 | -1.55 | 0.19 |
| Q79H | -1.100 | 18.774 | 0.638 | -0.838 | -0.51 | -0.947 | -0.22 | -0.931 | 0.72 | 0.23 |
| R359W | -1.400 | 23.690 | -0.204 | -0.391 | 0.163 | -0.132 | -0.32 | 0.448 | -0.05 | 0.02 |
| E339V | 0.800 | 21.855 | -0.074 | 0.264 | 0.059 | -0.31 | 1.5 | 0.47 | -0.48 | -1.05 |

**Table S6 -** Performance assessment of classification task prediction of effects of mutations on protein stability and flexibility for different structures of MAPK3.

| **MAPK3 – Inhibitor bound (4QTB)** | | | | | | | |
| --- | --- | --- | --- | --- | --- | --- | --- |
| **Method** | **TN** | **FP** | **FN** | **TP** | **MCC** | **F1** | **AUC** |
| DynaMut | 6 | 4 | 1 | 1 | 0.08 | 0.29 | 0.55 |
| DynaMut2 | 2 | 8 | 1 | 1 | -0.26 | 0.18 | 0.35 |
| mCSM | 0 | 10 | 0 | 2 | 0 | 0.29 | 0.50 |
| DUET | 2 | 8 | 1 | 1 | -0.26 | 0.18 | 0.35 |
| SDM | 2 | 8 | 0 | 2 | 0.20 | 0.33 | 0.60 |
| ENCoM | 0 | 10 | 0 | 2 | 0 | 0.29 | 0.50 |
| DDMut | 3 | 7 | 0 | 2 | 0.26 | 0.36 | 0.65 |
| **MAPK3 – bound to ATP (2ZOQ)** | | | | | | | |
| **Method** | **TN** | **FP** | **FN** | **TP** | **MCC** | **F1** | **AUC** |
| DynaMut | 4 | 0 | 4 | 4 | 0.50 | 0.67 | 0.75 |
| DynaMut2 | 1 | 3 | 1 | 7 | 0.16 | 0.78 | 0.56 |
| mCSM | 1 | 3 | 0 | 8 | 0.43 | 0.84 | 0.62 |
| DUET | 3 | 1 | 1 | 7 | 0.62 | 0.88 | 0.81 |
| SDM | 3 | 1 | 1 | 7 | 0.62 | 0.88 | 0.81 |
| ENCoM | 1 | 3 | 0 | 8 | 0.43 | 0.84 | 0.62 |
| DDMut | 0 | 4 | 4 | 4 | -0.50 | 0.50 | 0.25 |

**Table S7** - Effects of mutations on protein stability and flexibility for the CaM protein in its APO state (PDB: 1DMO).

| **Mutation** | **ΔS ENCoM** | **DUET** | **ENCoM** | **mCSM** | **SDM** | **DynaMut** | **DynaMut2** | **DDMut** |
| --- | --- | --- | --- | --- | --- | --- | --- | --- |
| N53I | -0.05 | 0.50 | 0.04 | 0.06 | 0.44 | 0.18 | 0.57 | 0.51 |
| F89L | 0.58 | -1.46 | -0.46 | -1.37 | -1.53 | -0.78 | -1.76 | -1.66 |
| D95H | 0.07 | 0.39 | -0.06 | 0.26 | 0.20 | -0.09 | 0.41 | 0.03 |
| D95V | 0.09 | 0.68 | -0.07 | 0.17 | 1.24 | 0.04 | -0.11 | 0.03 |
| N97I | -0.08 | 0.27 | 0.07 | 0.03 | 0.03 | -0.14 | 0.24 | 0.15 |
| N97S | 0.04 | 0.10 | -0.04 | 0.10 | -1.45 | -0.59 | 0.26 | 0.14 |
| A102V | -0.59 | -0.20 | 0.47 | -0.37 | -0.97 | 0.45 | -1.01 | -0.07 |
| E104A | 0.13 | -0.54 | -0.10 | -0.70 | -0.43 | -0.08 | 0.05 | -0.26 |
| N129G | 0.21 | 0.57 | -0.17 | -0.36 | 2.91 | 1.15 | 0.01 | -0.38 |
| N129V | 0.21 | 0.39 | -0.16 | 0.11 | 0.15 | 0.03 | 0.45 | -0.38 |
| D131E | 0.01 | -0.19 | -0.01 | -0.50 | 0.01 | 0.15 | 0.03 | 0.21 |
| D131H | -0.20 | 0.68 | 0.16 | 0.58 | 0.42 | 0.44 | 0.52 | 0.14 |
| D131V | 0.04 | 0.38 | -0.03 | 0.17 | -0.11 | -0.18 | -0.22 | 0.18 |
| D133H | 0.08 | 0.59 | -0.06 | 0.55 | 0.04 | 0.06 | 0.49 | 0.05 |
| Q135P | -0.02 | -0.20 | 0.02 | -0.15 | -1.21 | -0.10 | 0.10 | 0.53 |
| E140G* | 0.51 | -1.44 | -0.41 | -1.21 | -1.64 | -0.29 | -1.04 | -1.32 |

** excluded from the challenge*

**Table S8** - Effects of mutations on protein stability and flexibility for the CaM protein bound to Ca^2+^ (PDB: 1CLL).

| **Mutation** | **ΔS ENCoM** | **DUET** | **ENCoM** | **mCSM** | **SDM** | **DynaMut** | **DynaMut2** | **DDMut** |
| --- | --- | --- | --- | --- | --- | --- | --- | --- |
| N53I | 0.08 | 0.54 | -0.06 | 0.06 | 0.60 | -0.04 | 0.35 | 0.55 |
| F89L | 0.64 | -0.91 | -0.51 | -0.81 | -1.72 | -0.09 | -0.36 | -1.11 |
| D95H | 0.11 | -0.53 | -0.09 | -0.41 | -0.45 | -0.25 | -0.15 | 0.43 |
| D95V | 0.07 | -0.10 | -0.05 | -0.03 | -0.83 | -0.14 | 0.05 | 0.48 |
| N97I | -0.06 | 0.01 | 0.05 | -0.28 | 0.19 | 0.10 | -0.30 | 0.53 |
| N97S | -0.01 | -0.17 | 0.01 | -0.21 | -1.24 | -0.18 | 0.26 | 0.60 |
| A102V | -0.46 | -0.41 | 0.37 | -0.65 | -0.61 | 0.96 | -0.77 | 0.00 |
| E104A | 0.88 | -0.81 | -0.70 | -0.83 | -0.86 | -0.30 | -0.36 | -0.71 |
| D129G | 0.33 | -0.34 | -0.27 | -0.23 | -1.02 | 0.07 | 0.34 | -1.60 |
| D129V | -0.14 | 0.79 | 0.12 | 0.48 | 0.68 | 0.55 | 0.78 | -0.93 |
| D131E | 0.11 | -0.26 | -0.09 | -0.29 | -1.32 | -0.17 | -0.01 | 0.10 |
| D131H | -0.32 | -0.49 | 0.25 | -0.52 | -0.45 | 0.58 | -0.21 | -0.72 |
| D131V | 0.05 | -0.01 | -0.04 | -0.04 | -0.83 | 0.16 | -0.05 | -0.32 |
| D133H | 0.01 | -0.18 | -0.01 | -0.46 | 0.53 | -0.19 | -0.15 | -0.07 |
| Q135P | 0.49 | -0.14 | -0.40 | -0.11 | -1.04 | -0.50 | 0.24 | -1.76 |
| E140G* | 1.10 | -0.98 | -0.88 | -0.95 | -1.02 | -0.52 | -0.83 | -2.30 |

** excluded from the challenge*

**Table S9** - Description of structure-based methods used to predict changes in protein stability upon missense mutations.

| **Name** | **Description** |
| --- | --- |
| SDM | As a knowledge-based approach, SDM pioneered the use of conformationally constrained environment-specific substitution tables to calculate changes in thermal stability between wild-type and mutant protein. Webserver available at <http://structure.bioc.cam.ac.uk/sdm2>. |
| mCSM | mCSM was one of the first scalable computational tools to accurately predict the effects of mutations on protein stability and binding affinity. This approach consolidated our graph-based signatures framework and served as a powerful approach widely applied to the study of protein structure and mutational effects.  Webserver available at <https://biosig.lab.uq.edu.au/mcsm/>. |
| DUET | This method consolidates two complementary approaches (mCSM and SDM) in a consensus prediction, obtained by combining the results of the separate methods in an optimised predictor using Support Vector Machines (SVM).  Webserver available at <https://biosig.lab.uq.edu.au/duet/>. |
| ENCoM | The Elastic Network Contact Model employs a potential energy function and includes a pairwise atom-type non-bonded interaction term to add an extra layer of information regarding the effect of the specific nature of amino acids on dynamics within the context of Normal Mode Analysis. ENCoM tries to approximate ΔΔG through calculations of the vibrational entropy. Software available at <https://github.com/NRGlab/ENCoM>. |
| DynaMut | DynaMut implements a consensus estimate of changes in protein stability based on our graph-based signatures approach with dynamics properties calculated using NMA via Bio3D and ENCoM to generate an optimised and more robust predictor.  Webserver available at <https://biosig.lab.uq.edu.au/dynamut/>. |
| DynaMut2 | DynaMut2 incorporates information on protein dynamics and structural environment properties of wild-type residue with our graph-based signatures approach to provide an accurate prediction of mutation effects on stability and dynamics for single and multiple missense mutations.  Webserver available at <https://biosig.lab.uq.edu.au/dynamut2/> |
| DDMut | DDMut a fast and accurate siamese network to predict changes in ΔΔG of proteins upon single and multiple point mutations, leveraging both forward and reverse mutations to account for model anti-symmetry. Webserver available at <https://biosig.lab.uq.edu.au/ddmut> |

# 
